# Electrical Impedance Characterization of *in Vivo* Porcine Tissue Using Machine Learning

**DOI:** 10.2478/joeb-2021-0005

**Published:** 2021-11-20

**Authors:** Stephen Chiang, Matthew Eschbach, Robert Knapp, Brian Holden, Andrew Miesse, Steven Schwaitzberg, Albert Titus

**Affiliations:** 1 Department of Biomedical Engineering, University at Buffalo, The State University of New York, Buffalo, NY, USA; 2 Medtronic LLC Buffalo, USA; 3 Department of Surgery, University at Buffalo, The State University of New York Buffalo, NY USA

**Keywords:** Bioimpedance, surgical staplers, tissue characterization

## Abstract

The incorporation of sensors onto the stapling platform has been investigated to overcome the disconnect in our understanding of tissue handling by surgical staplers. The goal of this study was to explore the feasibility of *in vivo* porcine tissue differentiation using bioimpedance data and machine learning methods. *In vivo* electrical impedance measurements were obtained in 7 young domestic pigs, using a logarithmic sweep of 50 points over a frequency range of 100 Hz to 1 MHz. Tissues studied included lung, liver, small bowel, colon, and stomach, which was further segmented into fundus, body, and antrum. The data was then parsed through MATLAB's classification learner to identify the best algorithm for tissue type differentiation. The most effective classification scheme was found to be cubic support vector machines with 86.96% accuracy. When fundus, body and antrum were aggregated together as stomach, the accuracy improved to 88.03%. The combination of stomach, small bowel, and colon together as GI tract improved accuracy to 99.79% using fine k nearest neighbors. The results suggest that bioimpedance data can be effectively used to differentiate tissue types *in vivo*. This study is one of the first that combines *in vivo* bioimpedance tissue data across multiple tissue types with machine learning methods.

## Introduction

**Fig. 1:**
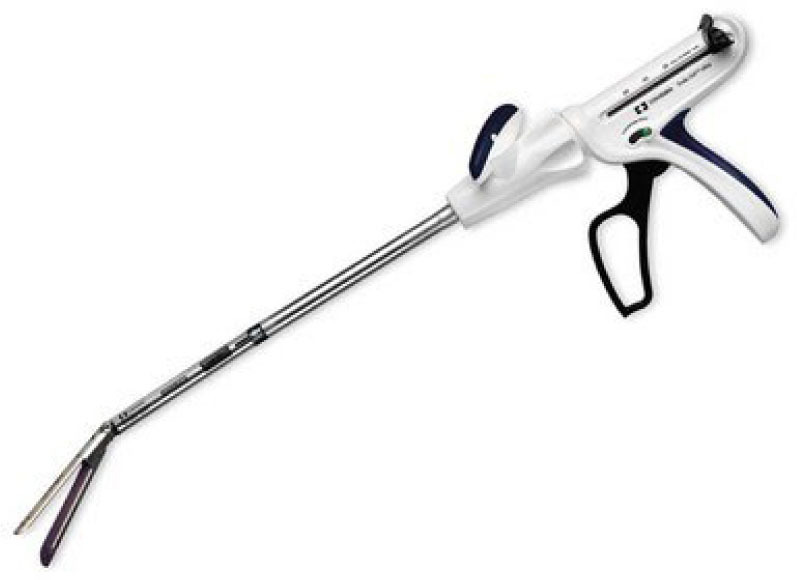
Medtronic's Endo GIA laparoscopic stapler

Surgical staplers are widely used in surgical procedures such as bariatric, thoracic, colorectal, and general surgery due to their versatility and efficiency (Fig. 1). However, each tissue type has its own challenges when stapling. For example, the stomach typically increases in thickness from the fundus down to the antrum and this must be accounted for when choosing the staple size to preserve staple line integrity and prevent leakage of gastric contents [1]. One of the drawbacks of endo-staplers that impacts stapling procedure is the loss of tactile feedback, which leads to a disconnect between surgeons and the tissue handling characteristics of these devices [2]. The incorporation of sensing technology within these instruments provides an opportunity for us to supplement, and even improve, our characterization of tissue handling in real time by enabling feedback control of the stapler. Multiple modalities of tissue characterization have been explored in the literature including, but not limited to optical [3], biomechanical [4], and bioelectric methods [5,6,7]. Electrical impedance has emerged as a promising tissue characterization modality as it offers good temporal resolution and has the advantages of being non-invasive and non-ionizing. However, it suffers from poor spatial resolution and is prone to artifacts from electrode movement and contact quality [8]. The utility of electrical impedance within medical instruments has already been demonstrated as advanced bipolar devices that deliver precise energy to seal and divide tissue by monitoring changes in tissue impedance have been on the market for over 20 years [9]. By advancing our understanding of tissue impedance further, we believe that bioimpedance can provide more sophisticated characterization beyond cauterized tissue to enable identification of tissue type and disease states.

Electrical impedance spectroscopy (EIS) is a method used to generate complex impedance measurements of dielectric materials, by inducing alternating current over multiple frequencies to the material. EIS can be applied to biological tissue to determine its pathological and physiological state. Impedance of biological tissues is dependent on cellular features such as intracellular and cell membrane contents. Broad architectural features such as cellular size, density, spacing, and constituents of the extracellular matrix (ECM) also play an important role [10]. Intracellular and extracellular fluids are ionic solutions that provide conducting paths for alternating currents and contribute to the resistance portion of impedance. Cell membranes are a lipid bilayer and contribute to a capacitive reactance [10]. The electrical response of tissues has been divided into different segments of frequency responses known as dispersions regions [11]. The α dispersion encompasses lower frequencies up to several kHz and is related to electrical conduction of the extracellular fluid. The β dispersion region ranges from several kHz to tens of MHz and involves the cell membrane and conduction of intracellular fluid. The γ dispersion region from MHz to GHz is associated with biological macromolecules such as proteins and organelles [12]. Electrical impedance has already been studied extensively as a means to distinguish benign and cancerous tissue. Multiple studies have demonstrated significant differences in impedance between normal and malignant tissue for liver, breast, and prostate [13,14,15]. Since tissue bioimpedance is highly dependent on the water content within tissue, studies have also explored using impedance to identify areas of hemorrhage or monitor ischemia [16, 17]. Several groups have also developed custom impedance probes and demonstrated success in differentiating tissue types in both ex-vivo [6] and in-vivo [18] settings.

The traditional method of representing bioimpedance data has been with modeling. An equivalent circuit to model bioimpedance of tissue was produced by the work of Cole *et al*. [19] and has been widely adopted for bioelectric characterization [20,21,22]. The Cole function, shown below, contains four parameters to model bioimpedance data,


$$Z\left(\omega \right)=R_{\infty }+\frac{R_{0}-R_{\infty }}{1+{\left(j\omega \tau \right)}^{\alpha }}$$

where ω is the angular frequency, R_0_ is the resistance at zero frequency, R_∞_ is the resistance at infinite frequency, α is a dimensionless number between 0 and 1 that describes the shape of the curve, and τ is a time constant corresponding to the characteristic frequency f_C_ (frequency at which the reactance is maximum) [13].

Machine learning (ML) in medicine has focused primarily on clinical imaging for feature extraction to streamline identification of cancer [23]. Improvements in algorithms and exponential growth in computing power have enabled analysis of large sets of high-dimensional data. While powerful, one of the major criticisms of ML is that machine-built models are often too complex for human understanding. The discrete relationships between variables can become muddled leading to a black box effect [24]. In general, machine learning (ML) has promise for tissue classification as increasingly large and complex data sets are generated. Expanded characterization efforts include not only simple tissue identification, but also disease states. Modeling methods have been effective in tissue impedance work when investigations have been limited in scope. However, when evaluating multiple tissue types and disease states, the complex pool of heterogenous data makes it increasingly difficult to correlate data to relevant equivalent circuit parameters, making ML more appealing and relevant. ML methods have been applied to bioimpedance data, albeit in a more limited scope. Several groups have applied machine learning algorithms to bioimpedance data in order to detect needle to nerve contact [25] and differentiate ischemic from healthy intestinal tissue *in vivo* [26]. But, to date, no one has performed classification of *in vivo* bioimpedance tissue data across multiple tissue types using ML methods.

In this work, we present and compare the results from *in vivo* electrical impedance measurements from pigs in order to attempt to use ML to classify healthy lung, liver, small bowel, colon, and stomach. To our knowledge, this is the first attempt at using ML to classify these *in vivo* tissue types based on EIS data.

## Materials and methods

**Fig. 2:**
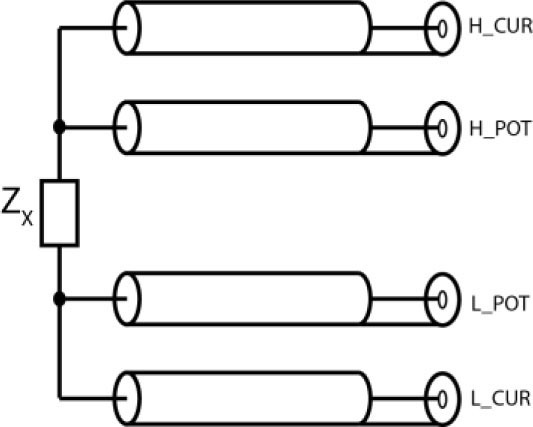
Electrode configuration. Z_x_ represents board impedance measurement. H_CUR and L_CUR are connected to ground electrodes, while H_POT and L_POT are the sense electrodes.

All impedance measurements were performed using a new, noncommercially available evaluation board (Analog Devices, Massachusetts, USA), based on but not identical to the AD5940. As per the manufacturer notes, the board utilizes a direct digital synthesis (DDS) chip that can set the test signal frequency in 0.2 Hz increments up to 10 MHz and change the amplitude with 16-bit resolution up to 2.4V. A customized flexible array strip (All Flex Inc) was designed with two rows 5 mm apart, each with ten gold leads 2.5 mm apart, mounted on a flexible copper strip. The sensor strip was interfaced with the module using a set of four co-axial cables. Impedance measurements in the literature have generally been performed with two or four electrode setups. For two electrode systems, the same pair of electrodes are used for both current injection and voltage measurement. As the alternating current is passed through the electrodes, contact impedance and electrode polarization gets added to the sample measurement which can lead to an over-estimation of tissue impedance [27]. While slightly more complex, the benefit of a four-electrode system is that measuring voltage with a very high input impedance prevents the flow of current in the sense electrodes [27]. Measurements in this study were performed using four-electrode testing arranged so that one pair of electrodes (outer electrodes) was used for current injection and another pair of electrodes (inner electrodes) was used for voltage measurement (see Fig. 2). This eliminates the electrode polarization impedance and allows the four-electrode system to perform more accurate measurements than two-electrode systems. All impedance measurements were obtained with a frequency sweep from 100 Hz to 1 MHz through fifty points, logarithmically. After each sweep, the measurements were exported as a data file. The data files were then exported and compiled into Excel using a custom MATLAB program.

**Fig. 3:**
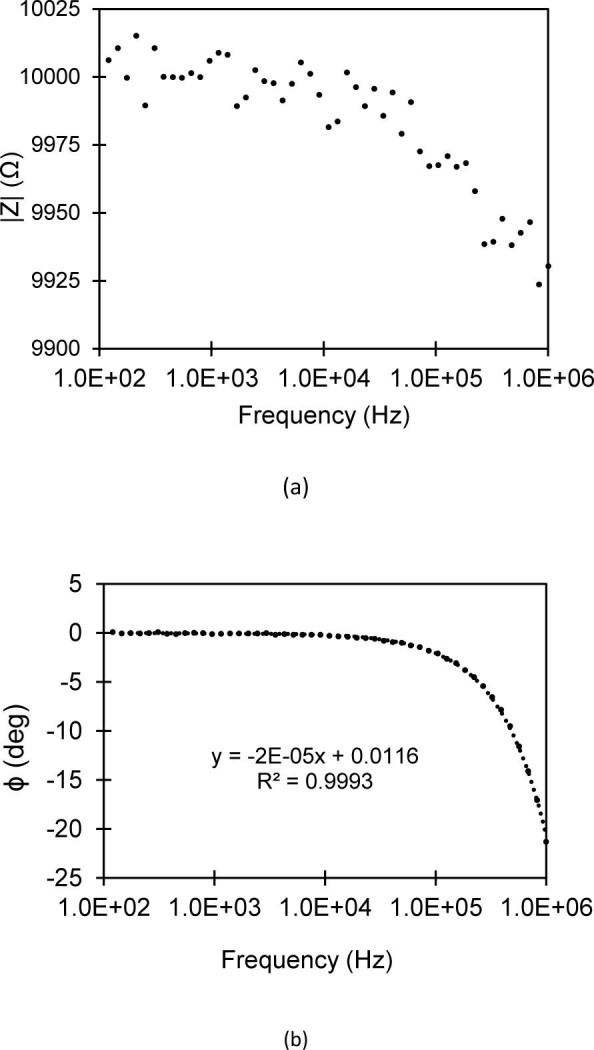
Validation of measurement system with known resistor values. (a) Modulus values of a control 10 kΩ resistor. (b) Phase angle values of a control 10 kΩ resistor. Phase angle measurements exhibit linear dependency on frequency with slope -2E-5.

The impedance measurement system was validated against multiple known resistors. The modulus and phase angle values obtained from a standard 10 kΩ resistor are depicted above in Fig. 3a and 3b. The modulus measurements show good accuracy with <1% error at the highest end of our testing frequency. The phase angle for an ideal resistor should be zero, but a linear dependency based on the frequency was identified in our system. There was a drift in the phase angle by -2E-5 degrees with each 1 Hz increase in frequency. At low frequencies, the phase angle remains low and essentially zero; however, this increases to a 20-degree difference at a frequency of 1 MHz. This difference can likely be attributed to capacitance within the measuring system. All subsequent measurements were adjusted accordingly to account for this phase angle frequency dependence.

### In Vivo Porcine Testing

For the *in vivo* porcine tissue testing, a total of seven live pigs were used for impedance measurements. Healthy young domestic female pigs with weights ranging between 30–40 kg were utilized for testing. The pigs were sedated, intubated, and all animal procedures were conducted under a protocol approved by the Institutional Animal Care and Use Committee (IACUC) at Medtronic. The impedance testing was often performed as a secondary study, but no destructive testing was performed prior to impedance measurements. The hemodynamic status of the pig was continuously monitored, and testing was terminated if there was concern of hemodynamic compromise or tissue ischemia.

**Fig. 4:**
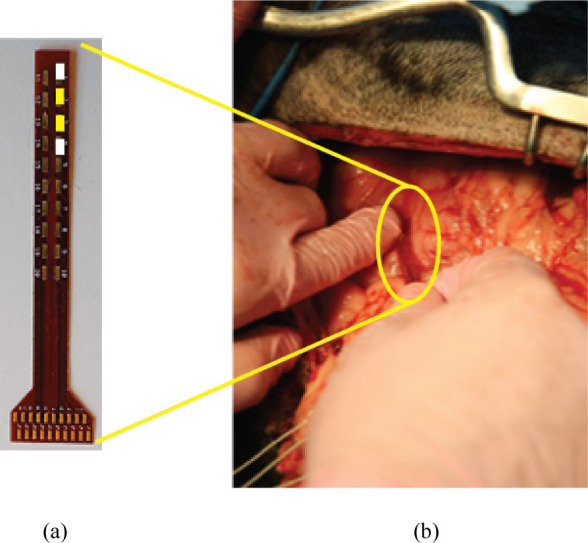
Electrode placement. (a) is the electrode array that is described in section IIA, and shown in Fig. 2. Four total electrodes are used. The top and bottom white electrodes are H_CUR and L_CUR and are connected to ground electrodes, while the center yellow electrodes are H_POT and L_POT, the sense electrodes. (b) The electrode array is manually applied to the tissue for measurements.

A midline laparotomy was initially performed. While pigs share similar metabolic and intestinal physiological processes with humans, and serve as good models for gastrointestinal tract, unlike humans, they have a larger and more developed cecum and the colon is arranged in a spiral structure [28]. The spiral colon was mobilized and attachments to itself were taken down to expose the colon for testing. The array strip (Fig. 4a) was manually placed against the tissue by an assistant ensuring that all four electrodes were in contact (Fig. 4b). The area of tissue examined by the electrode array is approximately 2 cm^2^. Impedance measurements were then obtained along different areas of the target tissue. The small bowel was then tested, followed by the liver. The attachments along the greater curve of the stomach were then taken down to mobilize the stomach for testing, making sure to maintain blood supply via the greater epiploic artery. Once all the intra-abdominal organs had been evaluated, a right thoracotomy was performed if feasible to provide exposure for lung testing. Due to a combination of lab availability constraints and animal instability, a thoracotomy was performed in only three pigs.

Across all seven pigs, a total of 106 impedance measurements were taken on the stomach. Of these measurements, 16 were clearly designated on the antrum, 20 on the body, and 14 on the fundus, while the remaining 56 measurements were broadly labeled as stomach without further delineation. There were 121 unique measurements on the colon, 82 on the liver, and 108 on the small bowel. Lung measurements were only able to be performed on three different pigs for a total of 33 measurements.

### Machine Learning

The validity of utilizing impedance for tissue differentiation was evaluated using the MATLAB classification learner machine learning tool. The resistance and reactance values for every tissue measurement were extracted from the data at seven frequencies: 145, 1400 11000, 49400, 184000, 570000, 1000000 for a total of fourteen parameters. The frequencies were arbitrarily chosen along a logarithmic scale based on previous experimental results to preserve the overall shape of the curve while also decreasing the processing burden. The models were run with 10-fold random cross-validation. Four methods were used to classify the data; these included (a) Decision Trees, (b) Support Vector Machines (SVM), (c) K-Nearest Neighbor Classifiers (KNN), and (d) Ensemble Classifiers. Each model was run ten times and the average accuracy and standard deviation was computed for each method and its associated classifier types. This was done to determine which method provided the most accurate characterization.

Details of the machine learning methods used in this study are available in detail from the MATLAB machine learning toolbox documentation [29], but are summarized here. Decision trees work by following the decisions in the tree from the root (starting) node as it splits down to a leaf node containing the response. Classifier types include coarse, medium, and fine which are determined by the maximum number of splits used (4, 20, and 100 respectively). As the number of splits increase, the likelihood of overfitting also increases. A fine tree with many leaves may be highly accurate on the training data but perform poorly when tested on an independent data set.

SVM classifies data by finding the hyperplane that optimizes the separation of data points between classes. The classifier type describes the shape of the hyperplane used to separate the data points. The classifier types for this method include linear, quadratic, cubic, and Gaussian which is further separated into fine, medium, and coarse. The kernel scale progressively decreases across the spectrum of coarse, medium, and fine Gaussian classifiers.

KNN works by categorizing a query point based on its distance to neighboring points in a training data set. The test point is assigned the label which is most frequent based on either a distance metric or set number of neighbors. Classifier types tested include fine, medium, coarse with increasing number of neighbors (1, 10, and 100, respectively). The classifiers set to 10 neighbors can be further subdivided into cosine, cubic, and weighted based on the structure of the distance metric. Nearest neighbor classifiers typically have good predictive accuracy in low dimensions but perform more poorly in higher dimensions.

Ensemble classifiers combine more than one machine learning algorithm into a predictive model. The classifier types are named based on the methods combined and include boosted trees, bagged trees, subspace discriminant, subspace KNN, and RUSBoosted trees.

### Ethical approval

The research related to animal use complied with all the relevant national regulations and institutional policies for the care and use of animals.

## Results

**Fig. 5:**
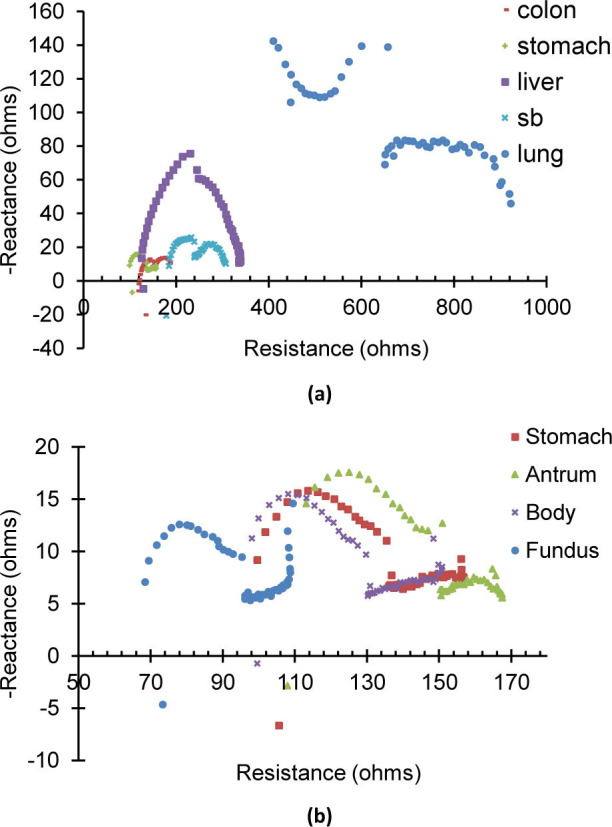
Nyquist plots for EIS measurements. (a) Comparison between all measured tissue types: colon, liver, small bowel, lung, and aggregate stomach. (b) Comparison between just stomach segments (fundus, body, antrum) and all stomach data taken as an aggregate.

A Nyquist plot is a common way to present impedance data where the real resistance portion of the impedance is plotted on the X-axis against the imaginary reactance portion along the Y-axis. The average values of the aggregate data were used to generate the Nyquist plot for each tissue and the results are presented in Fig. 5a for all tissues. From this, it is clear that the liver and lung Nyquist curves have distinctly unique shapes, differentiating them from the colon, stomach, and small bowel. The colon, stomach, and small bowel have similar shapes, however, their position along the plot allows for differentiation.

During stomach impedance measurements, the specific segment (fundus, body, antrum) was clearly delineated for about half of the measurements. The glandular architecture is different within the segments of the stomach and there is an increase in thickness progressing from the fundus to body to antrum [30]. Due to the differences in composition, the impedance measurements of each segment would also be expected to vary. The variation between different organs was much larger than the difference between stomach segments. The Nyquist plots for each of the stomach segments is shown in Fig. 5b and can be compared to the aggregate stomach data. The shape of the stomach, antrum, fundus, and body plots are identical, but the antrum appears skewed to the right while the fundus is skewed to the left. The antrum is the thickest portion of the stomach while the fundus is thinner and floppier, and this may provide a physiological explanation for our observations.

There was a consistent change in character of the phase angle at approximately 50 kHz for all tissue types. This may be attributed to represent the shift between α and β dispersion regions; however, prior work performed by Strand-Amundsen *et al*. on the bioelectrical characterization of *in vivo* porcine small bowel did not visualize the same shift at 50 kHz [31]. Their group utilized a different impedance measurement system (Solartron). As this shift is maintained throughout all tissue samples, the disparity is postulated to be related to the testing setup. Veal *et al*. described how capacitance of the coaxial cables that connect the electrodes to the circuit can provide a high frequency conduction path to ground, so that some of the current that passes through the sample bypasses the electrometer producing inductive artifacts [32].

**Fig. 6:**
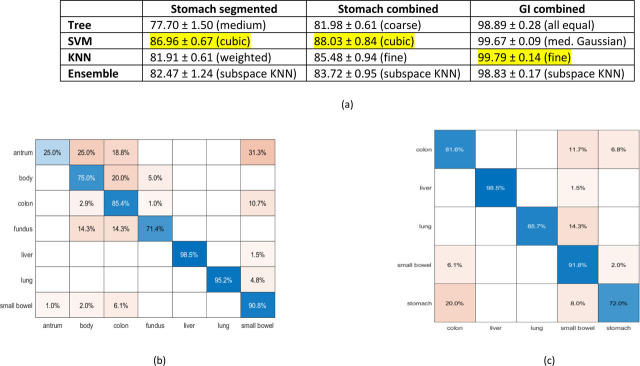
(a) Comparison of mean accuracy with standard deviations for the different machine learning classification methods, along with best performing subclass. Confusion matrix plots generated from a single 10 fold cross-validation run using the cubic support vector machine subclass with (b) stomach separated out into fundus, body, and antrum and (c) fundus, body, and antrum aggregated together as stomach prior to comparison to other tissues.

The data was parsed through the MATLAB classification learner three different ways: one where the stomach was segmented into body, fundus, antrum and compared to the small bowel, colon, liver, and lung; another where the stomach was taken as an aggregate; and finally, where the stomach was combined with the small bowel and colon to define GI tract structures and compared to the liver and lung. The mean accuracy and standard deviation along with the best performing subclass after applying the four different machine learning methods (decision trees, support vector machines, nearest neighbor classifiers, and ensemble classifiers) are presented in Fig. 6a. Support vector machines yielded the best results with accuracy of 86.96% when the stomach was segmented and 88.03% when stomach was taken as an aggregate. The antrum was the stomach segment that was most the difficult to identify with an accuracy of only 25% (Fig. 6b). When the fundus, body, and antrum are aggregated together as “stomach,” the stomach was the most frequently misclassified tissue, most often mistaken for the colon at 20% (Fig. 6c). When the stomach was combined with colon and small bowel to define the GI tract, all the algorithms had accuracy >98%, but fine KNN performed the best with 99.79% accuracy.

## Discussion

We recognize the limitation of this work due to the small dataset, but believe that the demonstration of this approach on actual tissue samples is important. Additionally, segmentation of the stomach divisions is limited compared to the small bowel and colon which have much larger surface areas for unique measurements. Future work is planned to test the classification ability of our model for unknown tissue samples from different pigs. Tissue properties have a major impact on stapling mechanics and technique. Suboptimal handling of tissue can increase the risk of complications such as anastomotic leaks. Lung tissue is uniquely challenging due to its heterogeneity. It is highly variable in the number of solid elements such as bronchi and vasculature and elastic elements such as alveoli [2]. These structural differences clearly differentiate lung tissue, and this was reflected in the observed bioimpedance profile. The stomach segments, colon, and small bowel all shared similarly shaped Nyquist curves and were positioned within the same vicinity on the plot. As a result, these tissues were commonly confused with each other during the ML classification. Within the segments of the stomach, the antrum was skewed in position compared to the fundus while maintaining the overall shape of the curve. It was difficult to differentiate the antrum from small bowel or colon, but it was never confused with the fundus. Identifying the various segments of stomach may provide clinical benefit in gastric resections as differences in gastric wall thickness have been postulated as a cause for staple line leaks [33]. The antrum, small bowel, and colon had comparable bioimpedance profiles which may be the result of similar architecture. Small bowel and colorectal tissue are much more uniform with similar stapling techniques for both tissues; however, if we can correlate tissue perfusion and tension with bioimpedance characteristics, modifications to current techniques can be made to reduce anastomotic leaks.

A benefit of impedance sensors is that they are typically lower profile compared to optical and mechanical sensors. This allows for easier incorporation onto pre-existing surgical instrumentation and stapling platforms. The electrode array strip used for this study was designed to provide flexibility with electrode arrangements and had the added benefit of fitting along the jaws of a surgical stapler. One of the short-comings of bioimpedance is that measurements are highly sensitive to both tissue inhomogeneity and electrode contact. The spacing between the electrodes affects the depth that the tissue is sampled, and further investigation remains to determine any discrepancies between superficial and transverse tissue measurements. Measurements within our study were obtained through manual application of the electrodes to the tissue surface and significant variation was seen between measurements even within the same tissue type. The amount of force and electrode-tissue contact was not standardized and compounded the variation between measurements. Additionally, the effect of compression on tissue impedance measurements remains poorly studied. Compression effects on impedance are different in *ex vivo* and *in vivo* settings, but the reason for this remains unclear [34]. Future work would focus on optimization of the testing setup to reduce contact variation as well as exploring the effects of tissue thickness and compression.

## Conclusion

The focus of this study was to explore how impedance can be combined with ML to characterize different tissues. The results from our *in vivo* testing have expanded on bioimpedance characterization of multiple *in vivo* tissues and clearly demonstrate the capabilities of bioimpedance and ML for classifying tissue. There were obvious bioelectric differences observed between liver, lung, and gastrointestinal tissue with almost 100% classification accuracy across all machine learning algorithms. This work shows that impedance can be used to create a classification algorithm to identify commonly stapled tissue such as colon, small bowel, liver, stomach, and lung *in vivo*. With continued improvement through additional data that improves training of the ML tools, it can be possible to explore tissue compression effects and better control firing forces of surgical staplers depending on tissue type.

The feasibility of identifying tissue ischemia and malignancy has been proven repeatedly, but typically limited to a single tissue type, so next steps in this work would be to optimize the data collection and explore the feasibility of differentiating tissue states (healthy, ischemic, and malignant) using ML. Also, *in vivo* disease state, particularly for human tissues, remains limited. With more data collection, we can then develop tissue specific firing algorithms to optimize the stapling process such as site selection, staple height, and firing speed to improve surgical outcomes.

## References

[1] Susmallian S GD, Barnea R, Raziel A (2017). Correct Evaluation of Gastric Wall Thickness May Support a Change in Staplers Size When Performing Sleeve Gastrectomy. The Israel Medical Association Journal: IMAJ.

[2] Chekan E, Whelan RL (2014). Surgical stapling device-tissue interactions: what surgeons need to know to improve patient outcomes. Med Devices (Auckl).

[3] Eriksson S, Nilsson J, Sturesson C (2014). Non-invasive imaging of microcirculation: a technology review. Med Devices (Auckl).

[4] Baker RS, Foote J, Kemmeter P, Brady R, Vroegop T, Serveld M (2004). The Science of Stapling and Leaks. Obesity Surgery.

[5] Cheng Z, Dall'Alba D, Foti S, Mariani A, Chupin T, Caldwell DG (2019). Design and Integration of Electrical Bioimpedance Sensing in Surgical Robotic Tools for Tissue Identification and Display. Front Robot AI.

[6] Rigaud B, Hamzaoui L, Frikha MR, Chauveau N, Morucci JP (1995). In vitro tissue characterization and modelling using electrical impedance measurements in the 100 Hz-10 MHz frequency range. Physiological Measurement.

[7] Ruiz-Vargas A, Ivorra A, Arkwright JW (2018). Design, Construction and Validation of an Electrical Impedance Probe with Contact Force and Temperature Sensors Suitable for in-vivo Measurements. Sci Rep.

[8] Adler A, Boyle A (2017). Electrical Impedance Tomography: Tissue Properties to Image Measures. IEEE Transactions on Biomedical Engineering.

[9] Karande VC (2015). LigaSure™ 5-mm Blunt Tip Laparoscopic Instrument. J Obstet Gynaecol India.

[10] Bera TK (2014). Bioelectrical Impedance Methods for Noninvasive Health Monitoring: A Review. J Med Eng.

[11] Martinsen OG, Grimnes SG, Schwan HP (2002). Interface phenomena and dielectric properties of biological tissue. Encycl Surf Colloid Sci.

[12] Ramírez-Chavarría RG, Sánchez-Pérez C, Matatagui D, Qureshi N, Pérez-García A, Hernández-Ruíz J (2018). Ex-vivo biological tissue differentiation by the Distribution of Relaxation Times method applied to Electrical Impedance Spectroscopy. Electrochimica Acta.

[13] Gregory WD, Marx JJ, Gregory CW, Mikkelson WM, Tjoe JA, Shell J (2012). The Cole relaxation frequency as a parameter to identify cancer in breast tissue. Medical Physics.

[14] Halter RJ, Hartov A, Heaney JA, Paulsen KD, Schned AR (2007). Electrical Impedance Spectroscopy of the Human Prostate. IEEE Transactions on Biomedical Engineering.

[15] Laufer S, Ivorra A, Reuter VE, Rubinsky B, Solomon SB (2010). Electrical impedance characterization of normal and cancerous human hepatic tissue. Physiological Measurement.

[16] Dzwonczyk R, Rio Cd, Brown DA, Michler RE, Wolf RK, Howie MB (2004). Myocardial electrical impedance responds to ischemia and reperfusion in humans. IEEE Transactions on Biomedical Engineering.

[17] Yang L, Zhang G, Song J, Dai M, Xu C, Dong X (2016). Ex-Vivo Characterization of Bioimpedance Spectroscopy of Normal, Ischemic and Hemorrhagic Rabbit Brain Tissue at Frequencies from 10 Hz to 1 MHz. Sensors (Basel).

[18] Dai Y, Du J, Yang Q, Zhang J (2014). Noninvasive electrical impedance sensor for in vivo tissue discrimination at radio frequencies. Bioelectromagnetics.

[19] Cole K, Curtis H (1944). Electrical physiology: Electrical resistance and impedance of cells and tissues, in Medical Physics.

[20] Gholami-Boroujeny S, Bolic M (2016). Extraction of Cole parameters from the electrical bioimpedance spectrum using stochastic optimization algorithms. Med Biol Eng Comput.

[21] Seoane F, Buendia R, Gil-Pita R (2010). Cole parameter estimation from electrical bioconductance spectroscopy measurements. Annu Int Conf IEEE Eng Med Biol Soc.

[22] Ayllon D, Seoane F, Gil-Pita R (2009). Cole equation and parameter estimation from electrical bioimpedance spectroscopy measurements - A comparative study. Annu Int Conf IEEE Eng Med Biol Soc.

[23] Bowen PK, Shearier ER, Zhao S, Guillory RJ, Zhao F, Goldman J (2016). Biodegradable Metals for Cardiovascular Stents: from Clinical Concerns to Recent Zn-Alloys. Advanced healthcare materials.

[24] Schmidt J, Marques MRG, Botti S, Marques MAL (2019). Recent advances and applications of machine learning in solid-state materials science. npj Computational Materials.

[25] Kalvoy H, Tronstad C, Ullensvang K, Steinfeldt T, Sauter AR (2017). Detection of needle to nerve contact based on electric bioimpedance and machine learning methods. Conf Proc IEEE Eng Med Biol Soc.

[26] Strand-Amundsen RJ, Tronstad C, Reims HM, Reinholt FP, Høgetveit JO, Tønnessen TI (2018). Machine learning for intraoperative prediction of viability in ischemic small intestine. Physiological Measurement.

[27] Chowdhury A, Ghoshal D, Bera T, Chakraborty B, Naresh M (2017). Comparison of two and four electrode methods for studying the impedance variation during cucumber storage using Electrical Impedance Spectroscopy (EIS).

[28] Gonzalez LM, Moeser AJ, Blikslager AT (2015). Porcine models of digestive disease: the future of large animal translational research. Transl Res.

[29] MathWorks Machine Learning Toolbox.

[30] Rawlins L, Rawlins MP, Teel D (2014). Human tissue thickness measurements from excised sleeve gastrectomy specimens. Surgical Endoscopy.

[31] Strand-Amundsen RJ, Tronstad C, Kalvøy H, Gundersen Y, Krohn CD, Aasen AO (2016). In vivo characterization of ischemic small intestine using bioimpedance measurements. Physiological Measurement.

[32] Veal B, Baldo P, Paulikas A, Eastman J (2015). Understanding Artifacts in Impedance Spectroscopy. Journal of the Electrochemical Society.

[33] Barski K, Binda A, Kudlicka E, Jaworski P, Tarnowski W (2018). Gastric wall thickness and stapling in laparoscopic sleeve gastrectomy - a literature review. Wideochir Inne Tech Maloinwazyjne.

[34] Moqadam S, Grewal P, Shokoufi M, Golnaraghi M (2015). Compression-dependency of soft tissue bioimpedance for in-vivo and in-vitro tissue testing. Journal of Electrical Bioimpedance.

